# Continuously Alternating Storage of Anion and Cation Toward a High‐Performance Bipolar Conjugated Polymer Cathode

**DOI:** 10.1002/advs.202503485

**Published:** 2025-06-23

**Authors:** Lian‐Wei Luo, Wenyan Ma, Siteng Zhu, Chong Zhang, Jia‐Xing Jiang

**Affiliations:** ^1^ Key Laboratory of Flexible Optoelectronic Materials and Technology (Jianghan University), Ministry of Education, School of Optoelectronic Materials & Technology Jianghan University Wuhan 430056 P. R. China; ^2^ School of Materials Science and Engineering Shaanxi Normal University Xi'an Shaanxi 710062 P. R. China; ^3^ Institute of Technology for Carbon Neutrality Shenzhen Institutes of Advanced Technology Chinese Academy of Sciences Shenzhen 518055 China

**Keywords:** bipolar conjugated polymers, ion co‐storage, organic cathode materials, rechargeable aluminum batteries

## Abstract

Rapid and sequential ion insertion is impeded in the single host organic electrodes, primarily due to Coulomb repulsion between ions with identical charges, which creates an irreconcilable conflict between capacity and rate performance. Herein, a bipolar conjugated donor‐acceptor (D‐A) polymer (PTZ‐Pz) is designed and synthesized with the co‐storage of opposite ions by utilizing the electron‐donating phenothiazine (PTZ) unit and the electron‐withdrawing phenazine (Pz) unit as the building blocks. It demonstrates that the continuously alternate storage of anions and cations can facilitate the charge transfer processes and thereby accelerate the reaction dynamic of the PTZ‐Pz cathode. Consequently, the D‐A polymer PTZ‐Pz shows a high specific capacity of 208 mAh g^−1^. The electrode exhibits an ultra‐long cyclability of more than 80 000 cycles, with an excellent rate performance of 116 mAh g^−1^ at 20 A g^−1^. Benefiting from the fast kinetics, the PTZ‐Pz cathode can operate well at a high mass loading of 44.2 mg cm^−2^. The enhanced energy and power density via alternate storage anions and cations mode may facilitate the exploration of new electrode materials for aluminum batteries.

## Graphical abstract

A bipolar D‐A polymer cathode is developed for aluminum dual‐ion batteries, the continuously alternate storage of anions and cations through Coulomb interactions enables the polymer cathode to achieve a high capacity of 208 mAh g^−1^, fast kinetics and long cyclability.

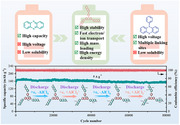

## 1 Introduction

The high abundance of aluminum in the Earth's crust and high theoretical capacity make rechargeable aluminum batteries (RABs) a viable and sustainable solution to the next‐generation energy storage technology.^[^ ^1^, ^2^, ^3^, ^4^, ^5^, ^6^ ^]^ To realize reversible stripping/plating of Al anode at room temperature, AlCl_3_‐based ionic liquids were widely used as the electrolytes in RABs, by virtue of their nonflammable and high ionic conductivity. Currently, one of the main challenges for RABs lies in the scarcity of high‐performance cathode materials that can reversibly host Al^3+^, since the high charge density of Al^3+^ results in sluggish diffusion kinetics in the cathode materials. Although numerous traditional cathode materials ranging from Prussian blue to chalcogenide and sulfide have been exploited in RABs,^[^ ^7^, ^8^, ^9^, ^10^ ^]^ it is still challenging for cathode materials to reversibly host Al^3+^ with a high capacity, low polarization, fast rate capability, and long cycle life. To obtain highly reversible electrochemical reactions in the cathode side of RABs. Dai groups utilized AlCl_4_^‐^‐hosting graphite as the cathode to assemble Al dual‐ion batteries with long cycle life, high rate, and high operation voltage, which provoked intense research on graphite‐based cathode materials.^[^ ^11^ ^]^ Unfortunately, conventional graphite cathodes suffer from limited specific capacity due to the single‐electron transfer with the storage of AlCl_4_^‐^ charge carriers.

Distinct from traditional inorganic materials, organic electrode materials have garnered recognition as promising candidates for rechargeable batteries, attributed to their controllable structure, cost‐effectiveness, and eco‐friendliness.^[^ ^12^, ^13^, ^14^, ^15^, ^16^ ^]^ Based on their reaction mechanisms, organic electrode materials are broadly classified into n‐type electrodes to store cations during charge/discharge processes and p‐type electrodes to host anions during the redox processes. Among the various organic electrode materials, n‐type conjugated imine‐based compounds have been proven to be high‐capacity electrode materials in rechargeable batteries.^[^ ^17^, ^18^, ^19^ ^]^ Imine‐based compounds typically have high intrinsic conductivity and fast reaction kinetics due to the presence of nitrogen atom with lone pair of electrons, while imine‐based small molecules commonly suffer from severe dissolution in ionic liquids electrolyte in RABs, leading to rapid capacity fading during repeated charge/discharge processes and low potential also limits its application. On the contrary, p‐type organic materials mainly include conductive polymers,^[^ ^20^ ^]^ quaternary ammonium,^[^ ^21^ ^]^ and polycyclic aromatic hydrocarbons.^[^ ^22^ ^]^ These materials are characterized by high redox potentials and fast redox kinetics based on p‐type doping/de‐doping reactions. However, their practical implementation faces a critical limitation—most p‐type organic cathodes exhibit relatively low specific capacities (<150 mAh g⁻¹), primarily due to the insufficient content of active groups.

By leveraging the established principles of reticular chemistry, the unique p‐type and n‐type organic building blocks can be organized into bipolar structures, allowing for the independent storage of cations and anions on the electroactive organic components to make the best of two types of organic electrodes.^[^ ^23^, ^24^ ^]^ During the polymerization process, various monomers can be grafted onto the molecular structures to achieve chemical design and modification.^[^ ^25^, ^26^ ^]^ Compared to n‐type or p‐type organic materials, bipolar organic materials possess both n‐type and p‐type redox active units as well as extended conjugated structures, promoting electron transfer and redox kinetics. Guided by this concept, it can be expected to obtain novel host materials that exhibit co‐storage of anions and cations, high capacity, and long cycling stability, thereby promoting the practical application of polymer cathodes for RABs.

With this in mind, we meticulously synthesized a n/p‐type bipolar conjugated donor–acceptor (D–A) polymer (PTZ‐Pz) cathode by integrating the electron‐donating phenothiazine (PTZ) unit with the electron‐withdrawing phenazine (Pz) unit into a crosslinked polymer framework. The crosslinked polymer skeleton could suppress the dissolution of both PTZ and imine‐based Pz units, contributing to long cycling life. The n/p‐type bipolar design is advantageous to the electrochemical kinetics of the resulting PTZ‐Pz cathode. Compared to the homopolymers PPTZ and PBPz, the PTZ‐Pz cathode with a donor–acceptor (D–A) molecular structure exhibits a narrower band gap and enhanced higher conductivity, which facilitates charge transport and expedites the reaction kinetics. Consequently, the PTZ‐Pz cathode demonstrates continuous alternating storage of anion (AlCl_4_^‐^) and cation (AlCl_2_^+^), which realized a four‐electron transfer during charging/discharging process and delivered an excellent electrochemical performance, including a high reversible capacity of 208 mAh g^−1^, excellent rate performance (116 mAh g^−1^ at 20 A g^−1^), ultra‐long cyclability over 80 000 cycles with minimal capacity decay. This work provides a new strategy for the development of high‐performance organic electrode materials through molecular design to realize high‐energy density RABs.

## 2 Results and Discussion

**Scheme 1:**
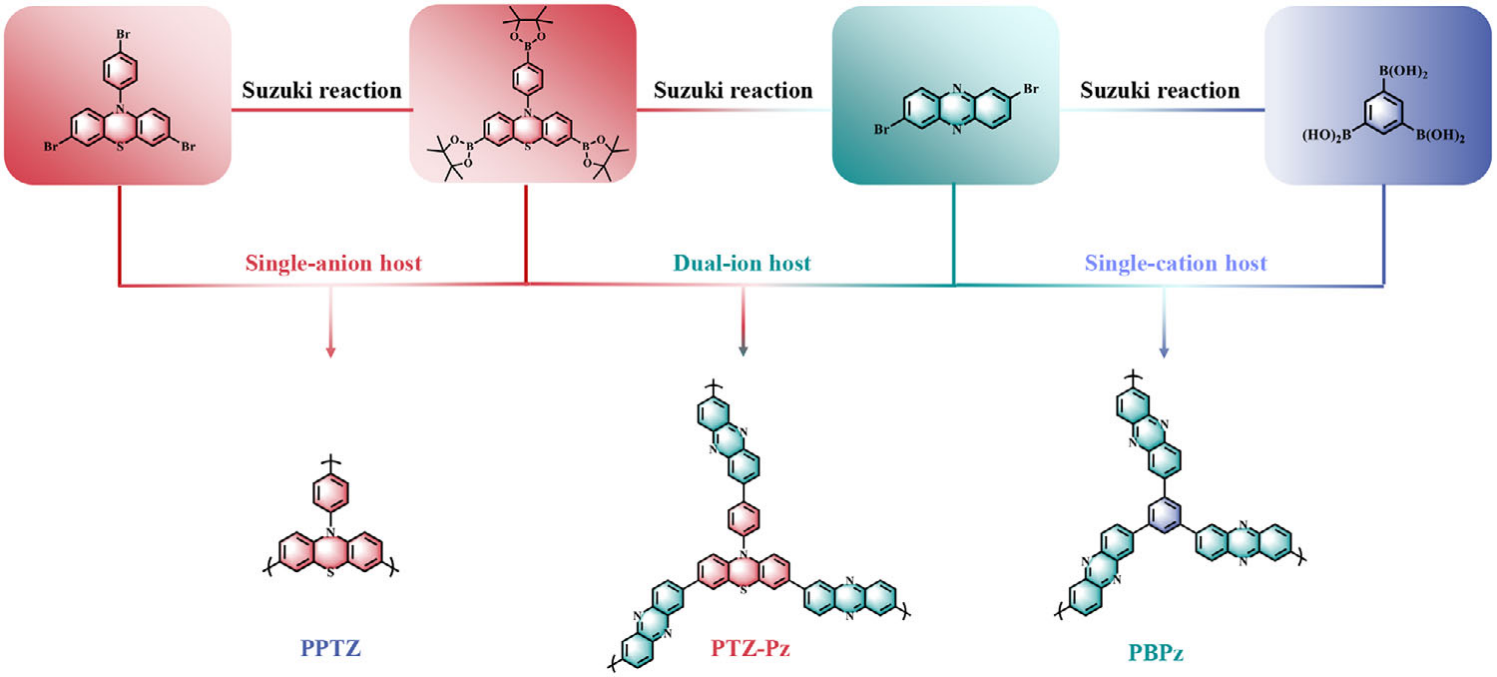
The synthetic routes for PPTZ, PBPz, and PTZ‐Pz with notional polymer structures.

**Figure 1:**
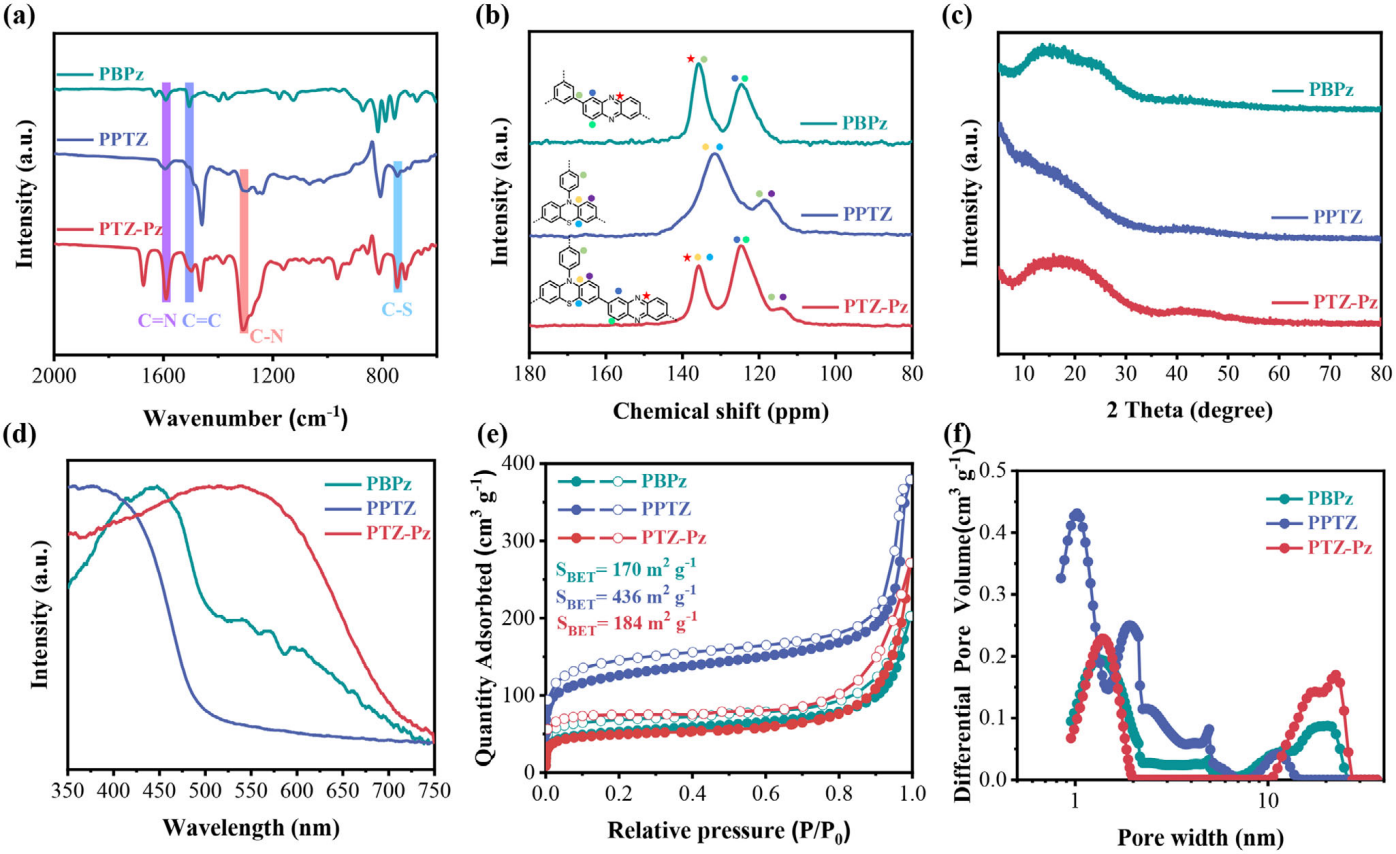
The characterization of PBPz, PPTZ, and PTZ‐Pz. a) The FT‐IR spectra. b) The solid‐state ^13^C NMR spectra. c) XRD patterns. d) UV–vis absorption spectra. e) Nitrogen adsorption (solid)‐desorption (hollow) isotherms. f) Pore size distribution curves obtained by NL‐DFT from the N_2_ adsorption data.

The bipolar polymer of PTZ‐Pz was synthesized via Suzuki‐Miyaura cross‐coupling reaction between brominated Pz (acceptor) and boronate‐functionalized PTZ (donor) (**Scheme** **1**; Figures S1, S2, Supporting Information). To delve into the impact of D‐A structure on the electrochemical performance, we also synthesized two other polymers of PBPz and PPTZ, which feature single redox‐active units of Pz (n‐type) and PTZ (p‐type), respectively. The chemical structure of the three polymers was confirmed by Fourier transform infrared (FT‐IR) spectrum and solid state ^13^C nuclear magnetic resonance (SS ^13^C‐NMR). As shown in **Figure** **1**a, the FT‐IR spectrum revealed the characteristic peak at 1595 cm^−1^ corresponding to the vibration of C═N bond in the Pz unit, and the peaks at ≈1300 and 746 cm^−1^ are attributed to C─N and C─S bonds in the PTZ unit, respectively.^[^ ^17^, ^27^, ^28^ ^]^ In the solid state ^13^C NMR spectrum (Figure 1b), the peaks at ≈135 ppm can be assigned to the C─N in both Pz and PTZ unit, and the peak at ≈124 ppm represents the carbon atom of the benzene ring, and the characteristic signal at ≈118 ppm belongs to the carbon atom bonded to S atom, which is absent in the ^13^C NMR spectrum of PBPz. Powder X‐ray diffraction (XRD) patterns demonstrated that all three polymers are non‐crystalline polymer structures (Figure 1c). Thermogravimetric analysis (TGA) showed that the D‐A structured PTZ‐Pz has a higher thermal stability than PBPz and PPTZ with an initial degradation temperature at ≈600 °C in the N_2_ atmosphere (Figure S3, Supporting Information). The scanning electron microscope (SEM) images showed that PTZ‐Pz has a nanosphere morphology aggregated from small nanosphere particles, PBPz exhibits a network of stacked nanoparticles, and PPTZ consists of nano‐sphere particles with diameters ranging from 200 to 400 nm (Figure S4, Supporting Information).

The ultraviolet–visible (UV–vis) spectrum showed that the D‐A polymer PTZ‐Pz has a wider light absorption range than the polymers of PBPz and PPTZ (Figure 1d), indicating a higher degree of conjugation in PTZ‐Pz, which might be assigned to its intrinsic electron “push–pull” effect from D‐A molecular structure.^[^ ^29^, ^30^, ^31^ ^]^ The porosity of the three polymers was analyzed by N_2_ sorption isotherms at 77.3 K. As shown in Figure 1e, all the polymers exhibit a mixture sorption isotherm of type I and III with a rapid nitrogen uptake at the low relative pressure (P/P_0_ < 0.001), suggesting the presence of micropores in all three polymers due to their highly crosslinked polymer structures. The surface area of 184 m^2^ g^−1^ for the D‐A polymer PTZ‐Pz lies between that of the single active polymers of PBPz (170 m^2^ g^−1^) and PPTZ (436 m^2^ g^−1^). According to nonlocal density functional theory (NL‐DFT), the pore size distribution curves were obtained from N_2_ adsorption data (Figure 1f). As shown in Figure 1e, PPTz has micropores centered at 1.01 nm, while PBPz and PTZ‐Pz show bigger micropores with diameters of 1.36 and 1.39 nm, respectively.

**Figure 2:**
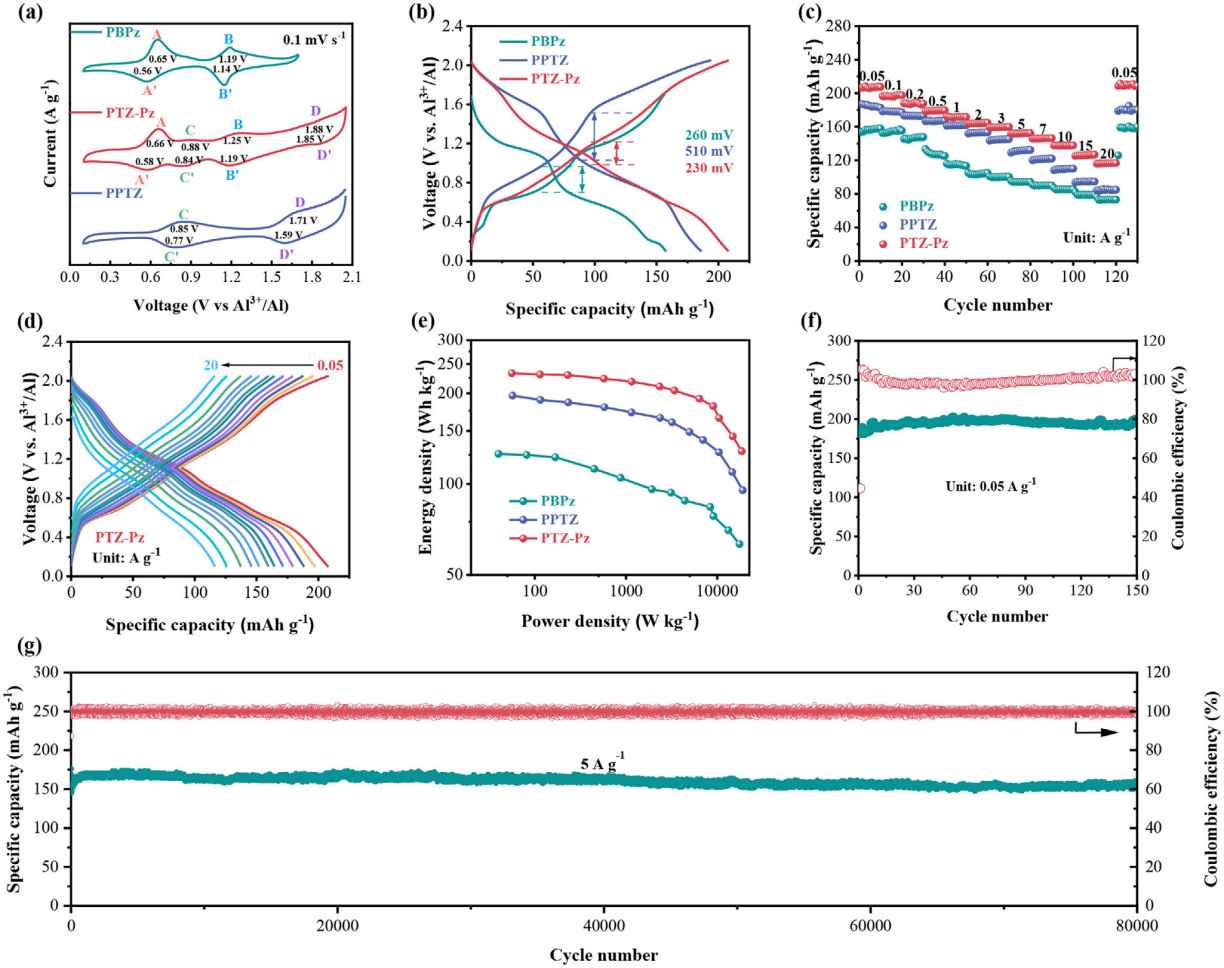
The electrochemical performance of PBPz, PPTZ, and PTZ‐Pz. a) CV curves at 0.1 mV s^−1^. b) the GCD curve at 0.05 A g^−1^. c) The comparison of rate performance. d) corresponding galvanostatic voltage profiles of PTZ‐Pz at current densities from 0.05 to 20 A g^−1^. e) Ragone plot of PBPz, PPTZ, and PTZ‐Pz battery, respectively. The cycling performance of PTZ‐Pz at f) 0.05 A g^−1^ and g) 5A g^−1^.

The electrochemical performance of the three polymer cathodes was investigated in a Swagelok‐type battery with an aluminum mesh anode and AlCl_3_‐based ionic liquid electrolyte. The charge‐storage behavior of the three polymers was first investigated using cyclic voltammetry (CV) at a scan rate of 0.1 mV s^−1^ (**Figure** **2**a). PBPz shows two pairs of redox peaks centered at 0.65/0.56 (peaks A/A') and 1.19/1.14 V (peaks B/B“), corresponding to the n‐type doping/dedoping reactions of C═N bond in the Pz unit, along with the insertion/de‐insertion of the cations. In the CV curve of PPTZ, two pairs of redox peaks were also observed at higher potentials 0.85/0.77 V (peaks C/C”) and 1.71/1.59 V (peaks D/D'), indicating the successive two‐step p‐type redox reactions of S and N atoms in the PTZ unit, which is consistent with previous research on aluminum or lithium dual‐ion batteries of PTZ‐based electrode.^[^ ^32^, ^33^ ^]^ As expected, the D‐A polymer PTZ‐Pz exhibits the characteristic redox peaks from both the Pz and PTZ units with a tiny change in the peak position, indicating the bipolar nature of PTZ‐Pz. The clear separation of the redox peaks in PTZ‐Pz confirms the alternating storage of cations and anions at different voltage ranges. In addition, PTZ‐Pz shows lower polarization during the redox reactions than the polymers of PBPz and PPTZ due to its smaller peak separation of the four pairs of redox peaks. Crucially, all redox potentials of PTZ‐Pz are higher than those of PPTZ and PBPz, likely due to its lower polarization of electrochemical reactions.

The galvanostatic charge/discharge (GCD) measurements revealed that the D‐A polymer PTZ‐Pz shows an impressive discharge capacity of 208 mAh g^−1^, much higher than that of the cation‐hosting PBPz (157 mAh g^−1^) and the anion‐hosting PPTZ (186 mAh g^−1^) (Figure 2b). PTZ‐Pz also has the lowest working overpotential of 230 mV among the three polymers, suggesting its low polarization. An outstanding rate performance was also obtained by PTZ‐Pz electrode, showing a highly reversible discharge capacity of 116.7 mAh g^−1^ at 20 A g^−1^ (Figure 2c,d). The excellent rate performance of PTZ‐Pz is attributed to its extended conjugation skeleton and intrinsic porous structure, which facilitate rapid electron and ion transport.^[^ ^34^, ^35^, ^36^ ^]^ In contrast, the PBPz and PPTZ cathode only exhibit a specific capacity of 73.2 and 84.9 mAh g^−1^, respectively, at a current of 20 A g^−1^ (Figure 2c; Figure S5, Supporting Information). The high capacity of PTZ‐Pz delivers a maximum energy density of 233 Wh kg^−1^ at a power density of 56 W kg^−1^, and a maximum power density of 18608 W kg^−1^ at 128.4 Wh kg^−1^ based on the mass of active material in the cathode (Figure 2e). Benefiting from the insoluble nature and intrinsic porous structure, PTZ‐Pz also exhibits an excellent cyclability with a capacity retention of 97.6% after 150 cycles at 0.05 A g^−1^ and maintains 92.3% capacity retention over 80 000 cycles at the current density 5 A g^−1^ (Figure 2f,g). Additionally, both PBPz and PPTZ also show stable cyclability with capacity retentions of 85% and 98% after 5000 cycles at 5 A g^−1^, attributed to their crosslinked structure and insoluble nature (Figure S6, Supporting Information). As far as we know, the high specific capacity, fast kinetics, and long cyclability of PTZ‐Pz represent the state‐of‐the‐art electrochemical performance of organic cathodes in AIBs (Table S1, Supporting Information).

**Figure 3:**
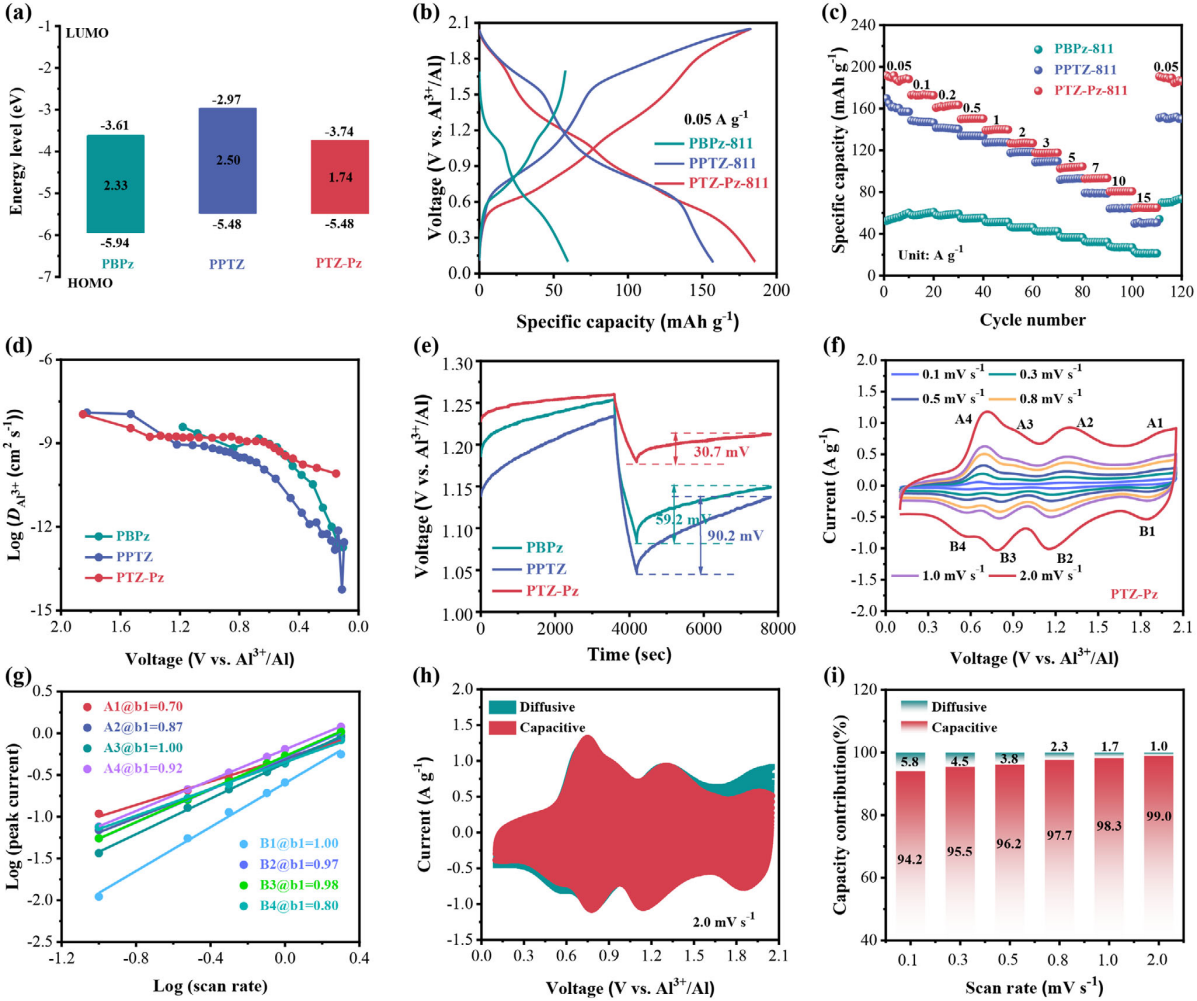
The band structures and electrochemical behavior of PBPz, PPTZ, and PTZ‐Pz. a) The band structures. b) The GCD curves of PBPz‐811, PPTZ‐811, and PTZ‐Pz‐811 c) The rate performance of PBPz‐811, PPTZ‐811, and PTZ‐Pz‐811. d) The diffusion rates of charge carriers in PBPz, PPTZ, and PTZ‐Pz. e). Comparison of the amplified GITT curves to show voltage relaxation process during discharge. f) The CV curves of PTZ‐Pz at the scan rates from 0.1 to 2 mV s^−1^. g) The fitted lines between log (*i*) and log (*v*) for PTZ‐Pz. h) Capacitive contribution of PTZ‐Pz at 2.0 mV s^−1^. i) Relative contributions of the capacitive and diffusion‐controlled charge storage of PTZ‐Pz at different scan rates.

To explain the structure‐performance relationships of the three polymers, we further evaluated their electronic and ionic transport properties. The band structures revealed that PTZ‐Pz exhibits a much narrower band gap (1.74 eV) compared to PPTZ (2.50 eV) and PBPz (2.33 eV) (**Figure** **3**a; Figure S7, Supporting Information). According to frontier molecular orbital theory, a narrow bandgap indicates high electronic conductivity. Therefore, the narrow bandgap of PTZ‐Pz implies its high electron conductivity due to its extended conjugation degree along the polymer chain arising from the D‐A structure.^[^ ^37^, ^38^ ^]^ To further confirm the impact of electron conductivity on the redox activity, we performed the control experiment by reducing the conductive additive content from 30 to 10 wt% in the three polymer cathodes. Consequently, the mass ratio of active materials, conductive carbon, and binder was adjusted from the original 6:3:1 to 8:1:1 (denoted as PBPz‐811, PPTZ‐811, and PTZ‐Pz‐811). GCD measurement revealed that the PTZ‐Pz‐811 cathode shows a lower capacity loss (22 mAh g^−1^) compared to PPTZ‐811 (29 mAh g^−1^) and PBPz‐811 (100 mAh g^−1^) (Figure 3b; Figure S8, Supporting Information). Furthermore, we also evaluated the rate performance of three polymers with 10 wt% conductive additive. Even at 10 A g^−1^, PTZ‐Pz‐811 could deliver a specific capacity of 81 mAh g^−1^, much higher than that of PBPz‐811 (27 mAh g^−1^) and PPTZ‐811 (64 mAh g^−1^). These results indicate that the conductive additive content has a smaller effect on the redox activity of PTZ‐Pz, partially due to its higher electronic conductivity.

The migratory behavior of ions in the host materials is primarily influenced by internal ionic interactions and the intrinsic properties of the polymer cathode, the galvanostatic intermittent titration technique (GITT) was utilized to investigate the ionic diffusion rates in the three polymer cathodes.^[^ ^39^ ^]^ The diffusion coefficient of both PBPz and PPTZ exhibits a monotonically decreasing trend during the discharge process, which conforms to the gradual increase of polarization predicted by the single ion storage mechanisms.^[^ ^40^, ^41^ ^]^ In contrast, the diffusion coefficient of the D‐A polymer PTZ‐Pz exhibits a stable platform at the intermediate discharge voltage ranging from 1.4 to 0.6 V, where alternate storage of anion and cation (Figure 3d; Figure S9, Supporting Information). This result indicates that the interaction between anion and cation favors ion insertion. In addition, the diffusion coefficient of D‐A structure polymer PTZ‐Pz (10^−8^ to 10^−11^ cm^2^ s^−1^) is significantly higher than that of the single‐ion‐type n‐type PBPz or p‐type PPTZ cathode during the discharge process, which also higher 1–3 orders of magnitude than that of the reported diffusion coefficient for organic Al‐ion cathodes.^[^ ^26^, ^42^, ^43^, ^44^, ^45^ ^]^ Further GITT detailed analysis revealed that the voltage relaxation in PTZ‐Pz (≈30.7 mV) is primarily governed by ion diffusion, reaching equilibrium faster than that in PBPz (≈59.2 mV) and PPTZ (≈90.2 mV) (Figure 3e). It verifies an improved ion kinetic in bipolar host cathode and highlights the positive effect of cation‐anion coulombic interactions on alternate ion storage in the D‐A polymer PTZ‐Pz.

The charge‐storage behavior was evaluated by CV measurement at scan rates ranging from 0.1 to 2.0 mV s^−1^ (Figure 3f; Figures S10–S12, Supporting Information). The PTZ‐Pz cathode displays distinct redox peaks that retained their original shape with minimal polarization even at high scan rates, indicating efficient and reversible charge‐storage reactions. According to the power‐law relationship *i* = a*v* ^b^, where *i* is the current response and *v* presents the scan rate, the b values were found to be 0.5 or 1.0, representing semi‐infinite linear diffusion or a capacitive process, respectively.^[^ ^46^, ^47^ ^]^ The fitted b values for the four pairs of redox peaks were close to 0.9 of PTZ‐Pz (Figure 3g; Figure S13, Supporting Information), implying that the charge storage mechanism in the PTZ‐Pz cathode belongs to surface‐controlled pseudocapacitive behavior with fast kinetics. The charge‐storage contribution from the diffusion‐controlled process and capacitive process were further analyzed and shown in Figure 3h,i and Figure S14 (, Supporting Information). Even at a low scan rate of 0.1 mV s^−1^, the capacitive contribution accounts for 94.2% of the total charge storage, which rapidly enhances to 99.0% at 2.0 mV s^−1^. Besides its high specific capacity, the charge‐storage behavior in PTZ‐Pz remains fast, likely due to its excellent ionic and electronic conductivity.

**Figure 4:**
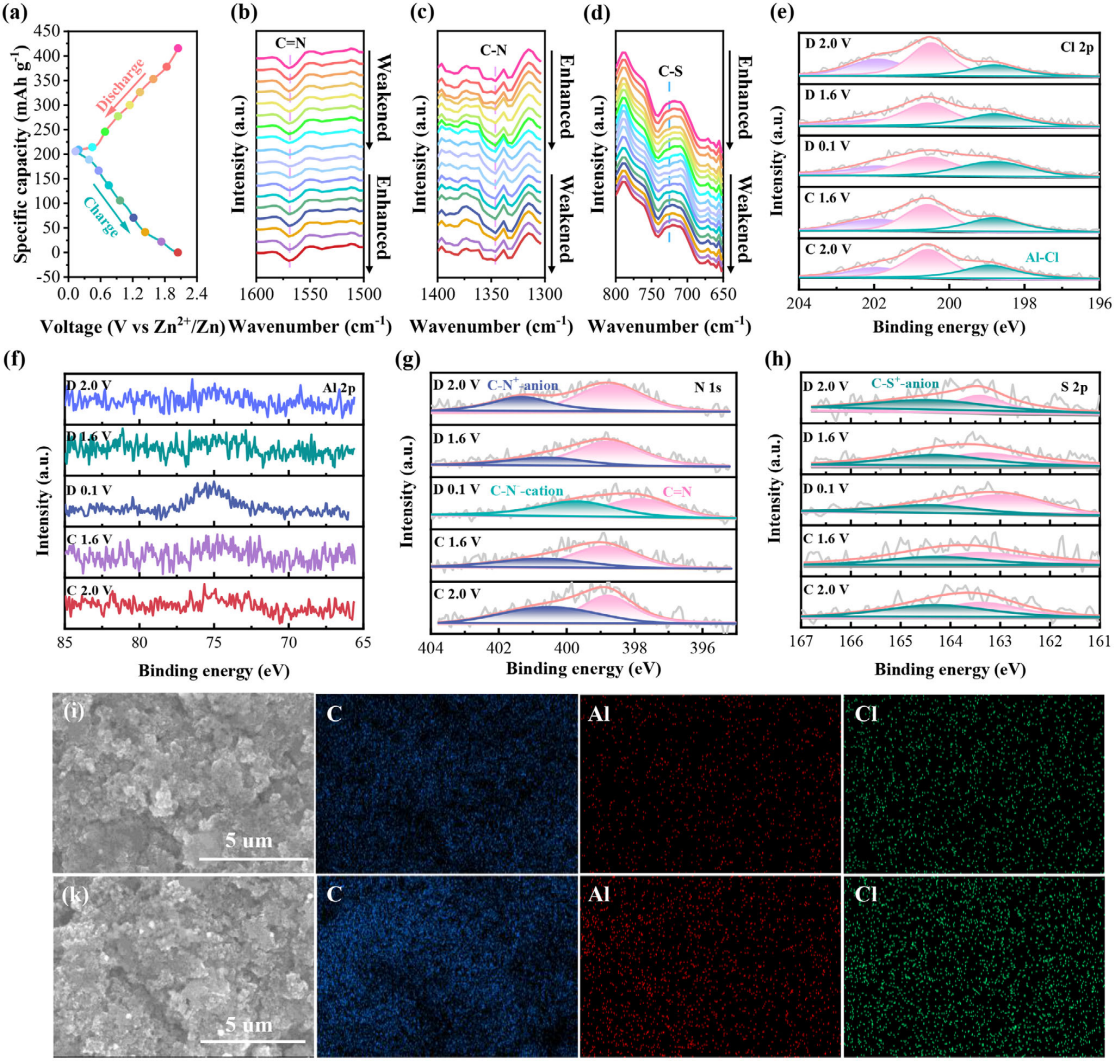
a) The GCD profiles of PTZ‐Pz at 0.05 A g^−1^. b–d) In situ FT‐IR spectra of the PTZ‐Pz cathode recorded at different charge–discharge states. The high‐resolution spectra of e) Cl 2p, f) Al 2p, g) N 1s, and h) S 2p for PTZ‐Pz cathode at different states (note: D means discharge, C means charge). The SEM images of the PTZ‐Pz cathode and the elemental mapping of C, Al, and Cl at fully i) discharged and k) charged states.

To obtain further comprehend into the charge‐storage mechanism of the polymer PTZ‐Pz cathode, In situ FT‐IR, ex situ X‐ray photoelectron spectroscopy (XPS), and EDX measurements were performed on the PTZ‐Pz cathode. The in situ FT‐IR spectra revealed that the absorption peak of C═N bond in Pz unit at ≈1569 cm^−1^ gradually diminished during the discharging process.^[^ ^48^ ^]^ However, the C─N and C─S bonds at 1346 and 726 cm^−1^ in PTZ units gradually enhanced during the discharge process. Conversely, during the charging process, the intensity of C═N bonds in the Pz unit gradually increases, while the C─N and C─S bonds in the phenothiazine (PTZ) unit progressively weaken. This behavior indicates a reversible switching of active sites in the PTZ‐Pz polymer (**Figure** **4b**‐‐d).^[^ ^26^, ^27^, ^28^ ^]^ To further elucidate the electrochemical reaction mechanism, ex situ XPS was utilized to analyze the evolution of the chemical state of the PTZ‐Pz cathode at different charge/discharge states. In high‐resolution XPS spectra, the peak intensity of Cl 2p signals weakened after the discharge process and then gradually strengthened upon the charge process, whereas the peak intensity of Al 2p signal displayed an opposite trend, which indicates that the organic ligand may interact with AlCl_4_^−^ anions or AlCl_2_^+^ cations through donating or accepting electrons due to its bipolar reactivity (Figure 4e,f).^[^ ^32^, ^49^, ^50^, ^51^, ^52^, ^53^, ^54^, ^55^ ^]^ The N 1s peak of PTZ‐Pz electrode was deconvoluted into three peaks at 397.8, 399.8, and 401.4 eV, corresponding to the C═N, C─N, and C─N^+^‐Al bonds, respectively. During the discharge process, first, the intensity of C─N^+^‐Al gradually weakened, indicating the anion release from C─N^+^, and then, as discharge progressed to 0.1 V, the intensity of C═N bond gradually decreased and the increasing intensity in C─N bond, this phenomena can be attributed to the reaction of cations with C═N bond in Pz unit.^[^ ^56^ ^]^ Subsequently, the C─N peak located at 399.8 eV gradually weakened during the charging process, signifying a reversible redox reaction in the Pz unit of PTZ‐Pz. The peak intensity at 401.4 eV increased with continued charging, indicating oxidation of the C─N bond and anion embedding in the PTZ unit (Figure 4g).^[^ ^57^ ^]^ In the S 2p spectra, the intensity of the peaks at higher binding energies increased after AlCl_4_^‐^ storage, indicating that the sulfur atom in the PTZ unit (‐S‐) was oxidized to positive charge (‐S^+^‐) (Figure 4h; Figure S15, Supporting Information).^[^ ^58^ ^]^ Given the bipolar charge‐storage characteristics, anions and cations function as the charge carriers during the charging and discharging processes in the PTZ‐Pz electrode, which is supported by the abundant Al and Cl signals observed in the elemental mapping images of both fully discharged and charged PTZ‐Pz (Figure 4i,k). The molar ratio of Cl to Al was found to be ≈4:1 in the fully charged state and 2:1 in the fully discharged state of PTZ‐Pz electrode (Figures S16, S17, Supporting Information), indicating that AlCl_4_^−^ and AlCl_2_^+^ simultaneously serve as the charge carriers for PTZ and Pz units in the bipolar polymer PTZ‐Pz.

**Figure 5:**
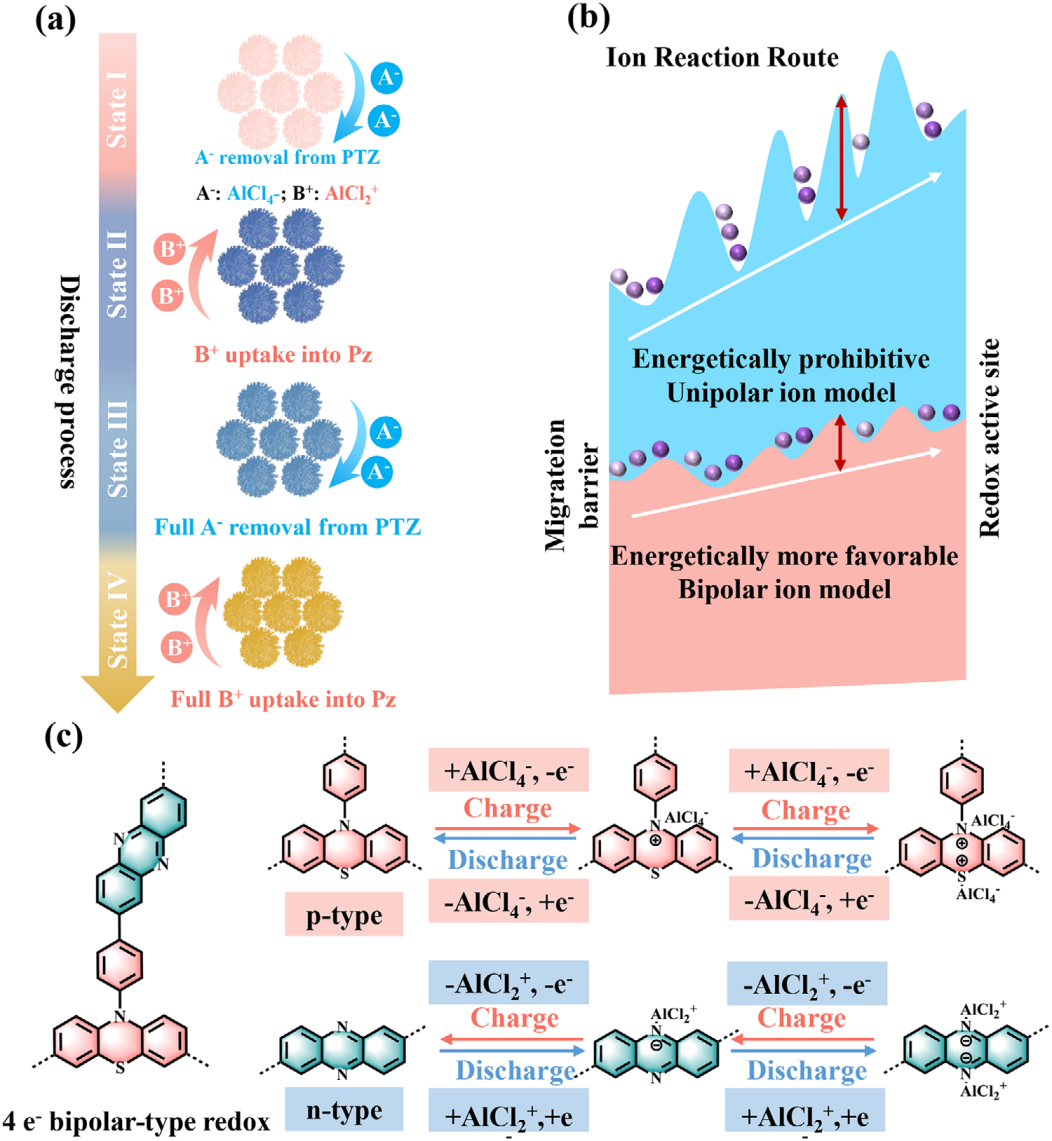
The charge storage mechanism of PTZ‐Pz. a) Schematic alternate storage of cation/anions in PTZ‐Pz cathode during the discharge process. b) Schematic ion migration routes toward organic redox‐sites based on unipolar cation or anion models (top), and bipolar cation–anion model (bottom). c) Redox reactions of PTZ‐Pz during the electrochemical process.

Spectroscopic and electrochemical analyses revealed that the bipolar PTZ‐Pz polymer undergoes a four‐step alternating charge storage process at multiple sites (**Figure** **5**a), which involves the reactions between AlCl_4_^‐^ with C─S and C─N bonds in the PTZ units, and AlCl_2_^+^ coordination with C═N bond in the Pz units. Figure 5b shows the reaction barrier landscapes of the single‐ion hosts (top) and bipolar hosts (bottom). In both PBPz and PPTZ, the rapid sequential migration of single ions is energetically unfavorable due to the presence of Coulombic repulsion between identical ions in increasingly crowded polymer chains, inhibiting charge storage. In contrast, the co‐storage mode of opposite ions in PTZ‐Pz is energetically more favorable, promoting multiple redox reactions at the C─N/C─S and C═N active sites, thus simultaneously increasing capacity and voltage. Therefore, we proposed that PTZ‐Pz cathode initiates consecutive four‐step single‐electron redox reactions, which entail primarily two‐step single‐electron redox reactions of C─N and C─S with AlCl_4_^‐^ in PTZ units and two‐step single‐electron redox reactions of C═N with AlCl_2_^+^ in Pz units (Figure 5c).

**Figure 6:**
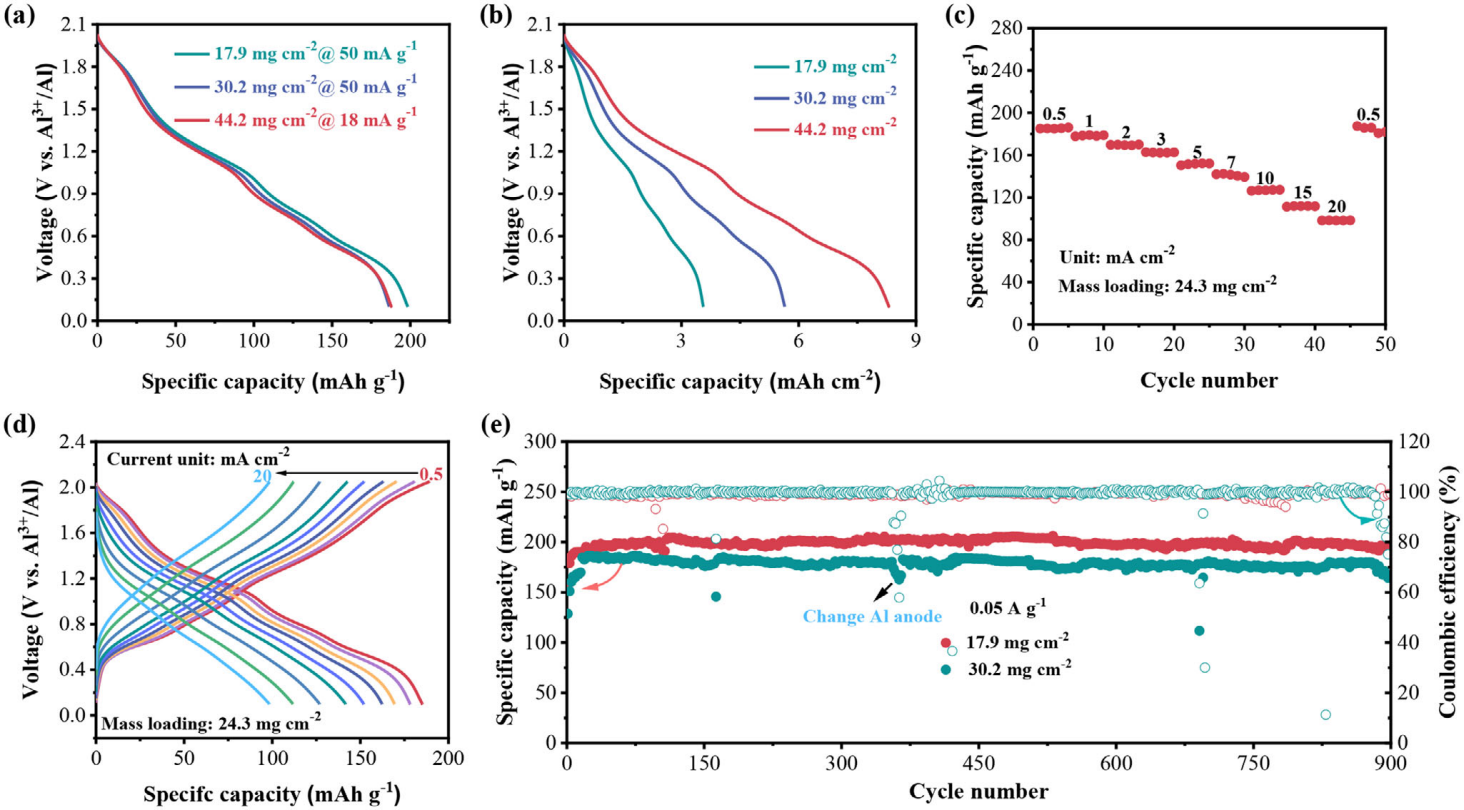
The electrochemical performance of PTZ‐Pz cathode with different mass loadings. The discharge curves at a) different current densities and b) mass loadings. c) The rate performance of PTZ‐Pz cathode with an active mass loading of 24.3 mg cm^−2^. d) The discharge curves of PTZ‐Pz at different currents from 0.5 to 20 mA cm^−2^. f) The cycling performance of PTZ‐Pz cathode with an active mass loading of 17.9 and 30.2 mg cm^−2^ at 0.05 A g^−1^.

Considering that increasing the mass content of electrochemically active materials can effectively enhance the energy density of batteries, it is essential for electrode active materials to operate at high mass loading. However, most redox‐active polymers exhibit obvious decay in their electrochemical performance after promoting their mass loadings, ascribed to their low ion and electron transportation kinetics.^[^ ^59^, ^60^ ^]^ Driven by the high redox activity and fast electrochemical kinetics, we further explored the potential of PTZ‐Pz at a high mass loading for practical applications. Notably, PTZ‐Pz exhibited only a slight capacity fade from 208 to 187 mAh g^−1^ as the mass loading of the active material increased from 1.5 to 44.2 mg cm^−2^. PTZ‐Pz achieved a specific capacity of 187 mAh g^−1^, corresponding to an areal capacity of ≈8.3 mAh cm^−2^ (**Figure** **6**a,b; Figure S18a, Supporting Information), which surpassing nearly all reported organic cathodes in RABs (Figure 6f; Table S2, Supporting Information). As depicted in Figure 6c, it is noteworthy that PTZ‐Pz cathode with a mass loading of 24.3 mg cm^−2^ was subjected to the rate test, it delivered high specific capacities of 185 and 98 mAh g^−1^, respectively, at current densities of 0.5 and 20 mA cm^−2^. Furthermore, the cycling stability of PTZ‐Pz with a mass loading of 17.9 and 30.9 mg cm^−2^ was rigorously evaluated at the current density of 0.05 A g^−1^. Remarkably, an areal capacity of 3.65 and 5.62 mAh cm^−2^ (corresponding to 198 and 186 mAh g^−1^, respectively) could be well maintained over 900 stable cycles (Figure 6e). Especially, even at a higher mass−loading of 44.2 mg cm^−2^, the PTZ‐Pz cell still can be cycled over 300 cycles with negligible capacity fading. Additionally, the cycling performance of PTZ‐Pz‐811 at a mass loading of 40.5 mg cm^−2^ was further assessed at the current density of 0.39 mA cm^−2^, which could maintain over 160 stable cycles (Figure S18b, Supporting Information). These findings clearly demonstrated that the exceptional electrochemical performance of PTZ‐Pz could be obtained even at high mass loadings, which is beneficial for practical applications.

## 3 Conclusion

In summary, we developed a bipolar D‐A polymer cathode for AIBs. The polymer features continuously alternate storage of anion (AlCl_4_^‐^) and cation (AlCl_2_^+^) in multiple active sites. The bipolar D‐A polymer combines the characteristics of n‐type and p‐type polymers, showing extended conjugation and narrow band gap, high ionic/electronic conductivities, and thus excellent electrochemical performance was achieved through a four‐electron transfer process during charging or discharging. The PTZ‐Pz cathode could deliver a high specific capacity of 208 mAh g^−1^, an outstanding rate performance with 116 mAh g^−1^ at 20 A g^−1^, and a long cyclability exceeding 80 000 cycles. Additionally, PTZ‐Pz cathode can also deliver a high areal capacity of 5.62 mAh cm^−2^ (186 mAh g^−1^) at an active mass loading of 30.9 mg cm^−2^, which could be well maintained over 900 stable cycles. Albeit the design of alternate storage of anions and cations could enhance the electron conductivity of PTZ‐Pz and thus enable PTZ‐Pz cathode to function well at high mass loadings and a relatively high conductive additive content (30 wt.%), it is still challenging for PTZ‐Pz cathode to realize high electrochemical performance at low conductive additive that is pivotal to the practical applications. Preparing PTZ‐Pz/conductive additive (e.g., low dimensional carbon materials) composites through in situ polymerization might be an efficient method to boost the electron conductivity and thus the electrochemical performance of PTZ‐Tz cathode with a low conductive additive content.

## 4 Experimental Section

Experimental details can be found in the Supporting Information.

## Conflict of Interest

The authors declare no conflict of interest.

## Author Contributions

L.‐W.L. and W.M. contributed equally to this work. L.‐W.L. performed methodology, validation, formal analysis, investigation, data curation, wrote the final manuscript. W.Y.M. performed formal analysis, investigation, wrote, reviewed, and edited the final manuscript. S.Z. performed formal analysis, investigation, wrote, reviewed, and edited the final manuscript. C.Z. performed resources, funding acquisition, wrote, reviewed, and edited the final manuscript. J.‐X.J. performed conceptualization, resources, supervision, funding acquisition, wrote, reviewed, and edited the final manuscript.

## Supporting information



Supporting Information

## Data Availability

The data that support the findings of this study are available from the corresponding author upon reasonable request.
