# Supplementary material for: Continuously Alternating Storage of Anion and Cation Toward a High‐Performance Bipolar Conjugated Polymer Cathode
Source: Adv Sci (Weinh). 2025 Jun 23;12(35):e03485. doi: 10.1002/advs.202503485 (PMC12462916; doi:10.1002/advs.202503485)
Supplement: Supplementary file 1 — Supporting Information [file ADVS-12-e03485-s001.docx]

***Supporting Information***

**Continuously Alternating Storage of Anion and Cation Towards A High-Performance Bipolar Conjugated Polymer Cathode**

Lian-Wei Luo^a,c‡^, Wenyan Ma^a‡^, Siteng Zhu^a^, Dan Li^a^, Chong Zhang^b^*, Jia-Xing Jiang^a^*

^a^Key Laboratory of Flexible Optoelectronic Materials and Technology (Jianghan University), Ministry of Education, School of Optoelectronic Materials & Technology, Jianghan University, Wuhan 430056, P. R. China.

^b^Key Laboratory for Macromolecular Science of Shaanxi Province, Shaanxi Engineering Laboratory for Advanced Energy Technology, School of Materials Science and Engineering, Shaanxi Normal University, Xi’an, Shaanxi 710062, P. R. China.

^c^Institute of Technology for Carbon Neutrality, Shenzhen Institutes of Advanced Technology, Chinese Academy of Sciences, Shenzhen 518055, China.

E-mail: jiaxing@jhun.edu.cn; [chongzhangabc@snnu.edu.cn](mailto:chongzhangabc@snnu.edu.cn)

**Experimental Procedures**

**Materials**

2,7-dibromophenazine, bis(pinacolato)diboron, anhydrous *N,N*-dimethylformamide (DMF), 10-phenyl-10H-phenothiazine, N-bromosuccinimide, 1,4-dioxane, benzene-1,3,5-triyltriboronic acid, [1,1’bis(diphenylphosphino)ferrocene]dichloropalladium(II) (Pd(dppf)Cl_2_), tetrakis-(triphenylphosphine)palladium(0) (Pd(PPh_3_)_4_) were purchased from TCI, Acros, Alfa, J&K and Yanshen Technology Co., Ltd., potassium carbonate, dichloromethane, acetic acid, ethyl acetate, petroleum ether, dichloromethane, acetone, toluene, tetrahydrofuran (THF) and methanol were purchased from Sinopharm Chemical Reagent Co. Ltd. All reagents were used as received. Aluminium chloride/1-ethyl-3-methylimidazolium chloride (EMImCl) ionic liquid (AlCl_3_/EMImCl=1.3) was purchased from DoDoChem.

**Synthesis**

*Synthesis of* *3,7-dibromo-10-(4-bromophenyl)-10H-phenothiazine*: N-Bromo-succinimide (10.69 g, 60.08 mmol) was added in five times to a solution of 10-Phenyl-10H-phenothiazine (5.0 g, 18.2 mmol) in THF (100 mL) and then stirred for 6 hours at 0-5 ℃. The mixture solution was poured into ice water and then filtered. Before drying under vacuum, the obtained residue was washed with water. The collected crude product was purified by column chromatography (petroleum ether/dichloromethane = 3/1) to obtain the target product (yield: 42.6%) as a white solid. 1H-NMR (400 MHz, CDCl_3_) δ 7.80-7.66 (m, 2H), 7.26-7.16 (m, 2H), 7.11 (d, J = 2.3 Hz, 2H), 6.94 (dd, J = 8.8, 2.3 Hz, 2H), 6.02 (d, J = 8.8 Hz, 2H).

**Fig. S1**. ^1^H NMR (a) and ^13^C NMR (b) spectra of 3,7-dibromo-10-(4-bromophenyl)-10H-phenothiazine in CDCl_3_.

*Synthesis of 3,7-bis(4,4,5,5-tetramethyl-1,3,2-dioxaborolan-2-yl)-10-(4-(4,4,5,5-tetramethyl-1,3,2-dioxaborolan-2-yl)phenyl)-10H-phenothiazine*: Under N_2_ atmosphere, the mixture of 3,7-dibromo-10-(4-bromophenyl)-10H-phenothiazine (2.56 g, 5 mmol), bis(pinacolato)diboron (4.57 g, 18 mmol), potassium acetate (3.54 g, 36 mmol) and Pd(dppf)Cl_2_ (0.27 g, 0.37 mmol) in dry 1,4-dioxane (50 mL) was stirred for 48 hours at 90 ^o^C. After cooling down to room temperature, the resulting suspension was extracted three times with ethyl acetate. The organic layers were combined, washed with saturated brine, dried with anhydrous Na_2_SO_4_ and filtered. A crude product was obtained after removing all the solvent by rotary evaporation, and further purified by quick chromatography using petroleum ether /EtOAc (15:1 v/v) as eluent (yield: 67.4%). 1H NMR (400 MHz, CDCl_3_) δ 8.04 (d, 1H), 7.44 – 7.31 (m, 2H), 7.18 (d, 1H), 6.04 (d, 1H), 1.40 (s, 6H), 1.28 (d, 12H).

**Fig. S2**. ^1^H NMR (a) and ^13^C NMR (b) spectra of 3,7-bis(4,4,5,5-tetramethyl-1,3,2-dioxaborolan-2-yl)-10-(4-(4,4,5,5-tetramethyl-1,3,2-dioxaborolan-2-yl)phenyl)-10H-phenothiazine in CDCl_3_.

*Synthesis of PPTZ*: Under the N_2_ atmosphere, aqueous potassium carbonate solution (2.0 M, 4 mL) was added to a solution of 3,7-dibromo-10-(4-bromophenyl)-10H-phenothiazine (1.0 mmol, 512 mg), 3,7-bis(4,4,5,5-tetramethyl-1,3,2-dioxaborolan-2-yl)-10-(4-(4,4,5,5-tetramethyl-1,3,2-dioxaborolan-2-yl)phenyl)-10H-phenothiazine (1.0 mmol, 653 mg) and tetrakis-(triphenylphosphine) palladium (0) (16 mg, 14 μmol) in DMF (20 mL). The mixture was heated to 150 ^o^C and stirred for 48 hours. After the reaction solution was cooled to ambient temperature and filtrated, the residue was washed with deionized water, methanol, dichloromethane and THF, respectively. The obtained polymer was further purified by *Soxhlet* extraction with tetrahydrofuran for 48 hours. Then the polymer was dried in a vacuum for 24 hours at 80 ^o^C to give a yellow-green powder (yield: 89.6%).

*Synthesis of the Polymer PBPz*: Under the N_2_ atmosphere, aqueous potassium carbonate solution (2.0 M, 4 mL) was added to a solution of 2,7-dibromophenazine (1.5 mmol, 507 mg), benzene-1,3,5-triyltriboronic acid (1.0 mmol, 210 mg) and tetrakis-(triphenylphosphine) palladium (0) (16 mg, 14 μmol) in DMF (20 mL). The mixture was heated to 150 ^o^C and stirred for 48 hours. After the reaction solution was cooled to ambient temperature and filtrated, the residue was washed with deionized water, methanol, dichloromethane and THF, respectively. The obtained polymer was further purified by *Soxhlet* extraction with tetrahydrofuran for 48 hours. Then the resulting polymer was dried in a vacuum for 24 hours at 80 ^o^C to give a pale green powder (yield: 85%).

*Synthesis of the Polymer PTZ-Pz*: Under the N_2_ atmosphere, aqueous potassium carbonate solution (2.0 M, 4 mL) was added to a solution of 3,7-bis(4,4,5,5-tetramethyl-1,3,2-dioxaborolan-2-yl)-10-(4-(4,4,5,5-tetramethyl-1,3,2-dioxaborolan-2-yl)phenyl)-10H-phenothiazine (1.0 mmol, 653 mg), 2,7-dibromophenazine (1.5 mmol, 507 mg) and tetrakis-(triphenylphosphine) palladium (0) (16 mg, 14 μmol) in DMF (20 mL). The mixture was heated to 150 ^o^C and stirred for 48 hours. After the reaction solution was cooled to ambient temperature and filtrated, the residue was washed with deionized water, methanol, dichloromethane and THF, respectively. The obtained polymer was further purified by *Soxhlet* extraction with tetrahydrofuran for 48 hours. The resulting polymer was dried in a vacuum for 24 hours at 80 ^o^C to give a red powder (yield: 87%).

**Electrochemical Performance Measurement**

The polymer cathodes were prepared by mixing the active material, carbon black and poly(vinylidenedifluoride) (PVdF) with a mass ratio of 6:3:1 or 8:1:1 in N-methyl 2-pyrrolidone (NMP) and then the mixture slurry was cast onto tungsten net, the electrodes were dried at 50 ^o^C for 5 hours in air and then dried at 90 ^o^C under vacuum for 12 hours. The mass loading of the active material is around 1.5 mg cm^−2^. For the preparation of PTZ-Pz cathode with high mass loadings (> 10 mg cm^-2^), PTZ-Pz, polytetrafluoroethylene (PTFE) binder, and isopropanol solvent with a specific mass ratio were mixed together in a 10 mL beaker. With the evaporation of isopropanol, the mixture became a soft monolith, which then was rolled into a freestanding film. After drying at 80 ^o^C for 12 h under vacuum, the freestanding cathode film was cut into pieces with a diameter of 10 mm for the battery assembling. The electrochemical measurements for the polymer cathodes were conducted in Swagelok-type cells with metallic aluminum anode, glass fiber (GF/D, Whatman) separator, and AlCl_3_:EMImCl ionic liquid (molar ratio: AlCl_3_/EMImCl=1.3) electrolyte. All the cells were half-cells because of the utilization of excess electrolyte and Al anode. For the cathodes with mass loadings higher than 10 mg cm^−2^, the PVdF binder and NMP solvent were replaced by polytetrafluoroethylene (PTFE) and isopropanol, respectively. Without specifying, the temperature for the electrochemical test was controlled in the range of 26~28 ^o^C. Of note, the rate performance was tested after cycling the battery for 50 cycles. The cyclic voltammetry measurement was conducted on an electrochemical workstation (CHI660E, Chenhua). The galvanostatic discharge-charge performance was performed on a LANHE CT2001A battery testing instrument. The electrochemical AC impedance spectrum was also evaluated in a CHI660E electrochemical workstation in the frequency ranging from 0.01 Hz to 100 kHz, and the cathode consisted of 80% active polymer, 10% conductive agent, and 10% binder. The calculated capacity was based on the mass of active materials. To eliminate the negative influence of Al dendrite on the cycling performance of the PTZ-Pz cathode with a mass loading of 17.9 mg cm^−2^, we respectively replaced the separator and Al anode at the 110^th^ and 770^th^ cycles during the cycling test.

**Characterizations**

The thermal stability of all the polymers was evaluated by a thermogravimetric analysis (Q1000DSC+LNCS+FACS Q600DT) with the temperature range from 20 to 800 ^o^C under N_2_ atmosphere. Fourier transform infrared (FT-IR) spectra were collected on transmission on a Tensor 27 FT-IR spectrometer (Bruker). The solid state ^13^C CP/MAS NMR spectra were collected on a JEOL RESONRNCE ECZ 400R NMR spectrometer with a MAS frequency of 12 kHz. The morphology of the polymers was obtained by a field emission scanning electron microscope (SEM) (SU8020, Hitachi). The specific surface area and pore size distributions were analyzed by an ASAP 2420-4 (micromeritics) volumetric adsorption analyze at 77.3 K. The surface area was calculated in the relative pressure (P/P_o_) range from 0.05 to 0.20. The pore size distribution data was calculated by NL-DFT utilizing the adsorption branch isotherm. The sample was degassed under vacuum (10^−5^ bar) at 120 ^o^C for 12 hours before N_2_ sorption analysis. Powder X-ray diffraction (PXRD) measurement was carried out on a X-ray diffractometer (D/Max-3c). X-ray photoelectron spectroscopy (XPS) measurement was performed on a ESCALAB Xi^+^ spectrometer (Thermo Fisher Scientific). The UV-vis reflection spectra were obtained on an UV-vis spectrophotometer (UV-Lambda 950, PerkinElmer, US) equipped with an integrating sphere assembly, using BaSO_4_ as a reflectance background. The CV measurement was carried out on a CHI660E (Chenhua, Shanghai) electrochemical workstation in a three-electrode-cell system, glassy carbon electrode as the working electrode, saturated calomel electrode as the reference electrode, platinum wire as the counter electrode. The ground polymer was mixed with 5 wt% Nafion, then the mixture was dropped cast on top of a glassy carbon working electrode and let the solvent evaporate in a vacuum chamber for 60 min. The measurement was carried out in a 0.1 M solution of tetrabutylammonium hexafluorophosphate (NBu_4_PF_6_) and acetonitrile with a scan rate of 100 mV s^-1^. The HOMO level (E_HOMO_) can be calculated by the equation of E_HOMO_=-(E_ox_–E_Fc/Fc_^+^+4.8), where E_ox_ is the standard oxidation potential (vs. standard hydrogen electrode (SHE)) of the polymer sample, E_Fc/Fc_^+^ is the redox potential (0.69 V vs. SHE) of ferrocene. Therefore, LUMO level (E_LUMO_) can be calculated by the equation of E_LUMO_ = E_HOMO_ + E_g_, where E_g_ is the band gap determined by the UV-vis absorption spectrum. For the *ex situ* XPS, and EDX measurements, the cells at different states of charge were disassembled in an argon-filled glove box and then the cathodes were washed by dried tetrahydrofuran several times for removing the residual electrolytes; Afterwards, the cathodes were dried under vacuum before the measurements. In the process of transferring the cathodes into the equipment, all samples were exposed to air within 5 s. For the *ex situ* FTIR measurement, the cells at different states of charge were disassembled in an argon-filled glove box, and the residual electrolytes on the surface of the cathodes were carefully wiped out, without using a washing process by solvents.

**Fig. S3**. Thermogravimetric analysis of PBPz, PPTZ and PTZ-Pz under N_2_ atmosphere.

**Fig. S4**. The SEM images of PBPz, PPTZ and PTZ-Pz.

**Fig. S5**. The GCD plots of (a) PBPz and (b) PPTZ at different current densities from 0.05 to 20 A g^-1^.

**Fig. S6**. The cycling performance of PBPz and PPTZ at (a) 0.05 A g^-1^, (b) 5 A g^-1^.

**Fig. S7**. The CV curves of the (a) PBPz, (b) PPTZ and (c) PTZ-Pz cathodes at 0.1V s^−1^.

**Fig. S8**. The GCD plots of PBPz-811, PPTZ-811 and PTZ-Pz-811 at different currents densities from 0.05 to 15 A g^-1^.

**Fig. S9**. The GITT response of the (a) PBPz, (b) PPTZ, (c) PTZ-Pz cathodes and (d) Schematic of GITT technique.

The diffusion of charge carriers of three polymers were quantified by galvanostatic intermittence titration technique (GITT). The electrode undergoes a series of current pluses for 10 min and is followed by a relaxation process for 60 min to reach equilibrium. The ionic diffusion coefficient (D) of active materials can be determined by the equation:

$D=\frac{4}{\pi\tau}\left( \frac{M_{b}V_{M}}{M_{B}S} \right)^{2}\left( \frac{\Delta E_{s}}{\tau(dE_{\tau}/D\sqrt{\tau}} \right)^{2} (\pi\ll L^{2}/D)$ (1)

$D=\frac{4}{\pi\tau}\left( \frac{V_{m}m_{B}}{M_{b}S} \right)^{2}\left( \frac{\Delta E_{s}}{{\Delta E}_{\tau}} \right)^{2}$ (2)

If the battery voltage is linearly related to τ^1/2^, the above formula can be simplified to the function (2):

Where τ is the current pulse time (s), V_m_ is the molar volume of polymer (cm^3^ mol^-1^), m_B_ is the mass of polymer in the electrode (g), M_B_ is the molar mass of polymer (g mol^-1^), S is the contact surface area (cm^2^) between electrode and electrolyte.

**Fig. S10**. The CV curves of PBPz and PPTZ at different scan rates.

**Fig. S11**. The capacitive contribution of the PBPz cathode at (a) 0.1, (b) 0.3, (c) 0.5, (d) 0.8, (e) 1.0 and (f) 2.0 mV s^−1^.

**Fig. S12**. The capacitive contribution of the PPTZ cathode at (a) 0.1, (b) 0.3, (c) 0.5, (d) 0.8, (e) 1.0 and (f) 2.0 mV s^−1^.

**Fig. S13**. The fitted lines between log (i) and log (v) for (a) PBPz and (b) PPTZ.

**Fig. S14**. The capacitive contribution of the PTZ-Pz cathode at (a) 0.1, (b) 0.3, (c) 0.5, (d) 0.8, (e) 1.0 and (f) 2.0 mV s^−1^.

**Fig. S15**. XPS survey spectra at different charge/discharge states.

**Fig. S16**. (a) The EDX data of PBPz cathode at fully discharged, (b) The EDX data of PPTZ cathode at fully charged.

**Fig. S17**. The EDX data of PTZ-Pz cathode at (a) fully discharged and (b) fully charged.

**Fig. S18**. (a) The cycling performance of PTZ-Pz cathode with a mass loadings of 44.2 mg cm^-2^ at 18 mA g^-1^. (b) The cycling performance of PTZ-Pz-811 cathode with a mass loadings of 40.5 mg cm^-2^ at 0.39 mA cm^-2^.

**Table S1**. The comparison of electrochemical performances of PTZ-Pz with most organic cathodes in rechargeable aluminum batteries (RABs) in recent investigations.

| **Samples** | **Charge**  **Carries** | **Specific capacity (mAh/g)**  **(current)** | **Rate capacity (mAh/g)**  **(current)** | **Cycle number (current)** | **Refs** |
| --- | --- | --- | --- | --- | --- |
| PTZ-Pz | AlCl_4_^-^  AlCl^2+^ | 208  (0.05 A/g) | 116.7  (20 A/g) | 150 (0.05 A/g)  80000 (5 A/g) | This work |
| TPB | AlCl_2_(urea)_2_^+^ | 180 (0.1 A/g) | 50 (1 A/g) | 250 (0.1 A/g) | 1 |
| COFJLU2 | AlCl_4_^-^ | 90 (2 A/g) | - | 30000 (5 A/g) | 2 |
| TCNQ | AlCl_2_^+^ | 120 (0.2 A/g) | 100 (0.5 A/g) | 2000 (0.5 A/g) | 3 |
| PAQS/MWCNTs | AlCl^2+^ | 165 (0.1125 A/g) | 133 (4.5 A/g) | - | 4 |
| H_2_TPP | AlCl_2_^+^ | 101.2 (0.1 A/g) | 74 (0.5 A/g) | 5000 (0.2 A/g) | 5 |
| poly(nitropyrene-co-pyrene) | AlCl_4_^-^ | 100 (0.2 A/g) | 48 (2 A/g) | 1000 (0.2 A/g) | 6 |
| PyPz | AlCl_4_^-^ | 231 (0.1 A/g) | 116 (30 A/g) | 100000 (10 A/g) | 7 |
| 2D Cu-based MOF@rGO | AlCl_2_^+^  AlCl_4_^-^ | 184 (0.05 A/g) | 65 (0.5 A/g) | 1000 (0.2 A/g) | 8 |
| PI@CNT | Al^3+^ | 130 (0.15 A/g) | - | 100  (0.15 A/g) | 9 |
| PHATN | AlCl_2_^+^ | 145 (0.05 A/g) | - | 100 (0.05 A/g) | 10 |
| anthracene | AlCl_4_^-^ | 157 (0.1 A/g) | 82 (0.5 A/g) | 800 (0.1 A/g) | 11 |
| PANI(H^+^)@SWCNTs | AlCl^2+^ | 200 (1 A/g) | 75 (40 A/g) | 8000 (10 A/g) | 12 |
| PyTQ-CNT | AlCl^2+^ | 208 (0.2 A/g) | 85 (2 A/g) | 4000 (1 A/g) | 13 |
| N4 | AlCl_4_^-^ | 136 (0.05 A/g) | 116 (2 A/g) | 4000 (1 A/g) | 14 |
| PVBPX | AlCl_4_^-^ | 133 (0.2 A/g) | 60 (10 A/g) | 50000 (5 A/g) | 15 |
| β-PT | AlCl_4_^-^ | 190 (2 A/g) | 127 (5 A/g) | 100000 (10 A/g) | 16 |
| MXene@BDTO | Al^3+^ | 229.8 (0.5 A/g) | - | 500 (0.5 A/g) | 17 |
| TpBpy-COF | AlCl_4_^-^ | 307 (0.1 A/g) | 113 (5 A/g) | 3000 (2 A/g) | 18 |
| PAQS/MWCNT | AlCl^2+^ | 160 (0.5 C) | 130 (10 C) | 500 (0.5 C) | 19 |
| X-PVMPT | AlCl_4_^-^ | 167 (0.5 C) | 64 (100 C) | 5000 (10 C) | 20 |
| PANI/OMC | AlCl_4_^-^ | 140 (0.1 A/g) | 118 (2 A/g) | 5000 (1 A/g)) | 21 |
| AQ | Al^3+^ | 215 (0.1 A/g) | 166 (0.5 A/g) | 200 (0.1 A/g) | 22 |
| TPBQ | AlCl^2+^ | 177 (0.02 A/g) | 122 (1 A/g) | 3000 (1 A/g) | 23 |
| PI/MOFs | AlCl^2+^ | 83 (1 A/g) | 40 (10 A/g) | 1800 (1 A/g) | 24 |
| PQ-Δ-HY | AlCl_2_^+^  AlCl_4_^-^ | 130 (0.1 A/g) | 92 (2 A/g) | 500 (0.2 A/g) | 25 |
| G-PANI-8 | AlCl_4_^-^ | 180 (1 A/g) | 155 (2 A/g) | 4000 (1 A/g) | 26 |
| TDK | AlCl^2+^ | 185 (0.1 A/g) | 66 (2 A/g) | 8000 (1 A/g) | 27 |
| PTh/MWCNT | AlCl_4_^-^ | 216 (0.0325 A/g) | - | 300 (0.13 A/g) | 28 |
| PTCDA-SP | Al^3+^ | 110 (0.1 A/g) | 72 (1000 A/g) | 1200 (0.1 A/g) | 29 |
| PNA | AlCl_2_^+^ | 178.2 (0.05 A/g) | 31.5 (10 A/g) | 10000 (2 A/g) | 30 |
| PTh/G | AlCl_4_^-^ | 152.5 (0.5 A/g) | 113.5 (1 A/g) | 500 (5 A/g) | 31 |
| PPP | AlCl_4_^-^  AlCl_2_^+^ | 92 (0.1 A/g) | 70 (10 A/g) | 30000 (10 A/g) | 32 |
| PTh@GO | AlCl_4_^-^ | 130 (1 A/g) | 86 (5 A/g) | 4000 (1 A/g) | 33 |
| C-PBPz | AlCl_4_^-^ | 145 (0.1 A/g) | 61 (20 A/g) | 80000 (10 A/g) | 34 |
| PPTZ-AQ | AlCl_4_^-^  AlCl^2+^ | 205 (0.05 A/g) | 160 (15 A/g) | 60000 (5 A/g) | 35 |

**Table S2**. The comparison of electrochemical performances between PPTZ-AQ and the reported organic cathodes with high active mass loadings in rechargeable aluminum batteries.

| **Samples** | **Mass Loading (mg/cm^2^)/Areal capacity (mAh/cm^2^)** | **Areal capacity (mAh/cm^2^)/Cylcle number** | **Refs** |
| --- | --- | --- | --- |
| PTZ-Pz | 17.9/3.65  44.2/8.3 | 3.65/1000 | This work |
| PyPz | 7.5/1.40  28.7/4.56 | 2.4/2000 | 7 |
| N4 | 6/0.77  12.9/1.61 | 0.768/50  1.61/50 | 14 |
| AQ | 12-16/2.58~3.44 | 2.58-3.44/200 | 22 |
| TBPQ | 5/0.71  9/1.09 | 0.71/200  1.09/200 | 23 |
| PQ-Δ-HY | 9/0.99 | 0.99/100 | 25 |
| PPP | 4/0.31  8/0.58 | 0.31/200  0.58/200 | 32 |
| C-PBPz | 7/0.88  45.1/4.51 | 4.51/50 | 34 |
| PPTZ-AQ | 72.3/14.24  100/17.20 | 5.1/580 | 35 |

**References**

1. Y. T. Kao, S. B. Patil, C. Y. An, S. K. Huang, J. C. Lin, T. S. Lee, Y. C. Lee, H. L. Chou, C. W. Chen, Y. J. Chang, Y. H. Lai, A quinone-based electrode for high-performance rechargeable aluminum-ion batteries with a low-cost AlCl_3_/urea ionic liquid electrolyte, *ACS Appl. Mater. Interfaces* 12 (2020) 25853-25860.
2. Q. Zhang, H. Wei, L. Wang, J. Wang, L. Fan, H. Ding, J. Lei, X, Yu, B, Lu, Accessible COF-based functional materials for potassium-ion batteries and aluminum batteries, *ACS Appl. Mater. Interfaces* 11 (2019) 44352-44359.
3. F. Guo, Z. Huang, M. Wang, W. L. Song, A. Lv, X. Han, J. Tu, S. Jiao, Active cyano groups to coordinate AlCl_2_^+^ cation for rechargeable aluminum batteries, *Energy Storage Mater*. 33 (2020) 250-257.
4. J. Bitenc, N. Lindahl, A. Vizintin, M. E. Abdelhamid, R. Dominko, P. Johansson, Concept and electrochemical mechanism of an Al metal anode‒organic cathode battery, *Energy Storage Mater*. 24 (2020) 379-383.
5. X. Han, S. Li, W. L. Song, N. Chen, H. Chen, S. Huang, S. Jiao, Stable high‐capacity organic aluminum–porphyrin batteries, *Adv. Energy Mater*. 11 (2021) 2101446.
6. M. Walter, K. V. Kravchyk, C. Böfer, R. Widmer & M. V. Kovalenko, Polypyrenes as high‐performance cathode materials for aluminum batteries, *Adv. Mater*. 30 (2018) 1705644.
7. W. Ma, L. W. Luo, X. Huang, P. Dong, Y. Chen, C. Zhang, F. Huang, J.-X. Jiang, Y. Cao, Dihydrophenazine‐based conjugated microporous polymer cathodes with enhanced electronic and ionic conductivities for high‐performance aluminum dual‐ion batteries, *Adv. Energy Mater*. 13 (2023) 2203253.
8. Y. Guo, W. Wang, H. Lei, M. Wang, S. Q. Jiao, Alternate storage of opposite charges in multisites for high‐energy‐density Al–MOF batteries, *Adv. Mater*. 34 (2022) 2110109.
9. X. Fan, F. Wang, X. Ji, R. Wang, T. Gao, S. Hou, J. Chen, T. Deng, X. Li, L. Chen, C. Luo, A universal organic cathode for ultrafast lithium and multivalent metal batteries, *Angew. Chem. Int. Ed*. 57 (2018) 7146-7150.
10. M. Mao, C. Luo, T. P. Pollard, S. Hou, T. Gao, X. Fan, C. Cui, J. Yue, Y. Tong, G. Yang, T. Deng, A pyrazine‐based polymer for fast‐charge batteries, *Angew. Chem. Int. Ed*. 58 (2019) 17820-17826.
11. D. Kong, T. Cai, H. Fan, H. Hu, X. Wang, Y. Cui, D. Wang, Y. Wang, H. Hu, M. Wu, Q. Xue, Polycyclic aromatic hydrocarbons as a new class of promising cathode materials for aluminum‐ion batteries, *Angew. Chem. Int. Ed*. 61 (2022) e202114681.
12. S. Wang, S. Huang, M. Yao, Y. Zhang & Z. Q. Niu, Engineering active sites of polyaniline for AlCl_2_^+^ storage in an aluminum‐ion battery, *Angew. Chem. Int. Ed*. 59 (2020) 11800-11807.
13. X. Peng, Y. Xie, A. Baktash, J. Tang, T. Lin, X. Huang, Y. Hu, Z. Jia, D. J. Searles, Y. Yamauchi, L. Wang, Heterocyclic conjugated polymer nanoarchitectonics with synergistic redox‐active sites for high‐performance aluminium organic batteries, *Angew. Chem. Int. Ed*. 61 (2022) e202203646.
14. G. Wang, E. Dmitrieva, B. Kohn, U. Scheler, Y. Liu, V. Tkachova, L. Yang, Y. Fu, J. Ma, P. Zhang, F. Wang, An efficient rechargeable aluminium–amine battery working under quaternization chemistry, *Angew. Chem. Int. Ed*. 61 (2022) e202116194.
15. Z. Yang, X. Huang, P. Meng, M. Jiang, Y. Wang, Z. Yao, J. Zhang, B. Sun, C. Fu, Phenoxazine polymer‐based p‐type positive electrode for aluminum‐ion batteries with ultra‐long cycle life, *Angew. Chem. Int. Ed*. 62 (2023) e202216797.
16. J. Zhang, Y. Wu, M. Liu, L. Huang, Y. Li, Y. Wu, Self‐adaptive re‐organization enables polythiophene as an extraordinary cathode material for aluminum‐ion batteries with a cycle life of 100 000 cycles, *Angew. Chem. Int. Ed*. 62 (2023) e202215408.
17. G. Wu, C. Lv, W. Lv, X. Li, W. Zhang, Z. Li, Anthraquinone derivatives supported by Ti_3_C_2_ (MXene) as cathode materials for aluminum-organic batteries, *J. Energy Chem*. 74 (2022) 174-183.
18. H. Lu, F. Ning, R. Jin, C. Teng, Y. Wang, K. Xi, D. Zhou, G. Xue, Two‐dimensional covalent organic frameworks with enhanced aluminum storage properties, *ChemSusChem* 13 (2020) 3447-3454.
19. N. Lindahl, J. Bitenc, R. Dominko & P. Johansson, Aluminum metal–organic batteries with integrated 3d thin film anodes, *Adv. Funct. Mater*. 30 (2020) 2004573.
20. G. Studer, A. Schmidt, J. Büttner, M. Schmidt, A. Fischer, I. Krossing, B. Esser, On a high-capacity aluminium battery with a two-electron phenothiazine redox polymer as a positive electrode, *Energy Environ. Sci*. 16 (2023) 3760-3769.
21. Y. Liao, D. Wang, X. Li, S. Tian, H. Hu, D. Kong, T. Cai, P. Dai, H. Ren, H. Hu, Y. Li, High performance aluminum ion battery using polyaniline/ordered mesoporous carbon composite, *J. Power Sources* 477 (2020) 228702.
22. L. Zhou, Z. Zhang, L. Cui, F. Xiong, Q. An, Z. Zhou, X. F. Yu, P. K. Chu, K. Zhang, High-capacity and small-polarization aluminum organic batteries based on sustainable quinone-based cathodes with Al^3+^ insertion, *Cell Reports Phys. Sci*. 2 (2021) 100354.
23. M. Mao, Z. Yu, Z. Lin, Y. S. Hu, H. Li, X. Huang, L. Chen, M. Liu, L. Suo, Simplifying and accelerating kinetics enabling fast-charge Al batteries, *J. Mater. Chem. A* 8 (2020) 23834-23843.
24. J. Zhou, X. Yu, J. Zhou, & B. Lu, Polyimide/metal-organic framework hybrid for high performance Al-organic battery, *Energy Storage Mater*. 31 (2020) 58-63.
25. D. J. Kim, D. J. Yoo, M. T. Otley, A. Prokofjevs, C. Pezzato, M. Owczarek, S. J. Lee, J. W. Choi, and J. F. Stoddart, Rechargeable aluminium organic batteries, *Nat. Energy* 4 (2019) 51-59.
26. D. Wang, H. Hu, Y. Liao, D. Kong, T. Cai, X. Gao, H. Hu, M. Wu, Q. Xue, Z. Yan, H. Ren, High-performance aluminum-polyaniline battery based on the interaction between aluminum ion and-NH groups, *Sci. China Mater*. 64 (2021) 318-328.
27. D.-J. Yoo, M. Heeney, F. Glöcklhofer, J. W. Choi, Tetradiketone macrocycle for divalent aluminium ion batteries, *Nat. Commun*. 12 (2021) 2386.
28. V. Raju, J. V. Rani & P. Basak, Self-arranged polythiophene on multi-walled carbon nanotube templated composites: synthesis and application in rechargeable aluminium battery, *Electrochim. Acta* 361 (2020) 137097.
29. L. Fang, L. Zhou, L. Cui, P. Jiao, Q. An, K. Zhang, Sulfur-linked carbonyl polymer as a robust organic cathode for rapid and durable aluminum batteries, *J. Energy Chem*. 63 (2021) 320-327.
30. K. Qin, S. Tan, M. Mohammadiroudbari, Z. Yang, X. Q. Yang, E. Hu, C. Luo, Synergy of carbonyl and Azo chemistries for wide-temperature-range rechargeable aluminum organic batteries, *Nano Energy* 101 (2022) 107554.
31. S. Zhao, H. Chen, J. Li, J. Zhang, Synthesis of polythiophene/graphite composites and their enhanced electrochemical performance for aluminum ion batteries, *New J. Chem*. 43 (2019) 15014-15022.
32. T. Li, H. Hu, T. Cai, X. Liu, Y. Zhang, L. Zhao, W. Xing, Z. Yan, Ultrafast and long-cycle stable aluminum polyphenylene batteries, *ACS Appl. Mater. Interfaces* 14 (2022) 30927-30936.
33. D. Kong, H. Fan, X. Ding, D. Wang, S. Tian, H. Hu, D. Du, Y. Li, X. Gao, H. Hu, Q. Xue, β-hydrogen of polythiophene induced aluminum ion storage for high-performance Al-polythiophene batteries, *ACS Appl. Mater. Interfaces* 12 (2020) 46065-46072.
34. W. Y. Ma, P. C. Zhang, L. T. Zhang, M. T. Ge, Y. P. Qi, Y. Chen, C. Zhang, J-X. Jiang, Towards durable and high-rate rechargeable aluminum dual-ion batteries via a crosslinked diphenylphenazine-Based conjugated polymer cathode, *ChemsusChem* 17 (2024) e202301725.
35. L-W. Luo, C. Zhang, W. Y. Ma, C. Z. Han, X. Ai, Y. Chen, Y. H. Xu, X. L. Ji, J-X. Jiang, Regulating the double-way traffic of cations and anions in ambipolar polymer cathodes for high-performing aluminum dual-ion batteries, *Adv. Mater*. 36 (2024) 2406106.
